# Major Pesticides Are More Toxic to Human Cells Than Their Declared Active Principles

**DOI:** 10.1155/2014/179691

**Published:** 2014-02-26

**Authors:** Robin Mesnage, Nicolas Defarge, Joël Spiroux de Vendômois, Gilles-Eric Séralini

**Affiliations:** ^1^University of Caen, Institute of Biology, CRIIGEN and Network on Risks, Quality and Sustainable Environment MRSH-CNRS, Esplanade de la Paix, 14032 Caen Cedex, France; ^2^CRIIGEN, 40 rue Monceau, 75008 Paris, France

## Abstract

Pesticides are used throughout the world as mixtures called formulations. They contain adjuvants, which are often kept confidential and are called inerts by the manufacturing companies, plus a declared active principle, which is usually tested alone. We tested the toxicity of 9 pesticides, comparing active principles and their formulations, on three human cell lines (HepG2, HEK293, and JEG3). Glyphosate, isoproturon, fluroxypyr, pirimicarb, imidacloprid, acetamiprid, tebuconazole, epoxiconazole, and prochloraz constitute, respectively, the active principles of 3 major herbicides, 3 insecticides, and 3 fungicides. We measured mitochondrial activities, membrane degradations, and caspases 3/7 activities. Fungicides were the most toxic from concentrations 300–600 times lower than agricultural dilutions, followed by herbicides and then insecticides, with very similar profiles in all cell types. Despite its relatively benign reputation, Roundup was among the most toxic herbicides and insecticides tested. Most importantly, 8 formulations out of 9 were up to one thousand times more toxic than their active principles. Our results challenge the relevance of the acceptable daily intake for pesticides because this norm is calculated from the toxicity of the active principle alone. Chronic tests on pesticides may not reflect relevant environmental exposures if only one ingredient of these mixtures is tested alone.

## 1. Introduction

Pesticides are used throughout the world as mixtures called formulations. They contain adjuvants, which are often kept confidential and are called inerts by the manufacturing companies, plus a declared active principle (AP), which is the only one tested in the longest toxicological regulatory tests performed on mammals. This allows the calculation of the acceptable daily intake (ADI)—the level of exposure that is claimed to be safe for humans over the long term—and justifies the presence of residues of these pesticides at “admissible” levels in the environment and organisms. Only the AP and one metabolite are used as markers, but this does not exclude the presence of adjuvants, which are cell penetrants. Our previous investigation showed unexpected APs for human cell toxicity in the adjuvants of glyphosate-based herbicides [1]. Ethoxylated adjuvants found in glyphosate-based herbicides were up to 10.000 times more toxic than the so-called active AP glyphosate [1] and are better candidates for secondary side effects. This may explain in vivo long-term toxicity from 0.1 ppb of the formulation and other toxicities that were not explained by a consideration of glyphosate alone [2–5]. These adjuvants also have serious consequences to the health of humans and rats in acute exposures [6, 7]. These findings prompted us to investigate the presence of similar toxic molecules in other classes of pesticides.

The regulatory system assumes that the AP designed to specifically target plants, insects or fungi is the most toxic compound of a formulation to nontarget species. Thus long-term regulatory tests are performed on this substance alone. In this paper, we tested to what extent the AP or adjuvants in present formulations account for the toxicity of 9 major pesticides: 3 herbicides, 3 insecticides, and 3 fungicides.

We have thus selected 9 APs of herbicides, insecticides, or fungicides of different classes (Table 1) used for agricultural or domestic purposes, from the major pesticides used worldwide [8, 9]. First we tested Roundup and its AP, glyphosate. Upon the introduction of herbicide tolerant genetically modified organisms (GMOs), designed to tolerate Roundup and to accumulate unusual levels of its residues, Roundup quickly became the major pesticide in the world and a major food or feed contaminant [10]. Two other herbicides of a different class were tested: isoproturon (phenylurea) is the second most widely used AP of herbicides in Europe in the control of annual grasses and broad-leaved weeds in cereals and a major water contaminant [11]; and fluroxypyr (a synthetic auxin) is used as an AP on noncrop areas and also for agricultural use on wheat, barley, corn, and oats. Forest services are expanding its use as an alternative to other pesticides known to be toxic [12]. However, it is poorly studied and its effects on human cells were never published before. Among the insecticides chosen, pirimicarb (a carbamate), used specifically to target aphids, is the most representative AP in this family for cereal production and garden insect control worldwide [13]. Neonicotinoids are the largest selling insecticides worldwide and are marketed in more than 120 countries for use on more than 140 crops [14]. Their spectrum of biological efficacy covers a broad range of target pests such as whiteflies, lepidopteran, and coleopteran species. We tested the major neonicotinoid, the AP imidacloprid, which is widely used for seed dressing. Its toxicity against bees is widely admitted [15], but little is known about the effects of its adjuvants. We also tested the AP acetamiprid, another neonicotinoid advocated to replace imidacloprid [16]. Azole-type fungicides are applied every year on field crops, fruit trees, vegetables, and grassgrowing areas [17]. We tested the two most popular triazole APs, epoxiconazole and tebuconazole. Finally, prochloraz (imidazole) was tested because it is the main fungicide sprayed on cereals in Europe [8].

We used the embryonic (HEK293), placental (JEG3), and hepatic (HepG2) human cell lines because they are well characterized and validated as useful models to test toxicities of pesticides [18–20], corresponding to what is observed on fresh tissue or primary cells [21–23]. These cell lines are even in some instances less sensitive than primary cells [24, 25] and therefore do not overestimate cellular toxicity. We assayed their mitochondrial succinate dehydrogenase (SD) activity (MTT assay) after 24 h pesticide exposure, which is one of the most accurate cytotoxicity assays for measuring the toxicity of pesticide adjuvants such as surfactants [26]. Cytotoxicity was confirmed by the measurement of apoptosis and necrosis, respectively, by caspases 3/7 activation [27] and adenylate kinase leakage after membrane alterations [28]. Each AP was tested from levels below its ADI to its solubility limit in our system. The formulations containing adjuvants were tested at the same levels.

## 2. Materials and Methods

### 2.1. Chemicals

The 9 Aps, glyphosate (N-phosphonomethyl glycine, G, CAS: 1071-83-6), isoproturon (3-(4-isopropylphenyl)-1,1-dimethylurea, CAS: 34123-59-6), fluroxypyr 1-methylheptyl ester (((4-Amino-3,5-dichloro-6-fluoro-2-pyridinyl)oxy)acetic acid, 1-methylheptyl ester, CAS: 81406-37-3), acetamiprid (N-[(6-chloro-3-pyridyl) methyl]-N′-cyano-N-methyl-acetamidine, CAS: 135410-20-7), imidacloprid (1-((6-chloro-3-pyridinyl)methyl)-4,5-dihydro-N-nitro-1H-imidazol-2-amine, CAS: 105827-78-9), pirimicarb (2-dimethylamino-5,6-dimethyl-4-pyrimidinyl dimethylcarbamate, CAS: 23103-98-2), prochloraz (N-propyl-N-(2,4,6-trichlorophenoxy)ethyl-imidazole-1-carboxamide, CAS: 67747-09-5), epoxiconazole (1-{[3-(2-Chlorophenyl)-2-(4-fluorophenyl)-2-oxiranyl]methyl}-1H-1,2,4-triazole, CAS: 135319-73-2), tebuconazole (1-(4-Chlorophenyl)-4,4-dimethyl-3-(1,2,4-triazole-1-ylmethyl)pentane-3-ol, CAS: 107534-96-3), and 3-(4,5-Dimethylthiazol-2-yl)-2,5-diphenyl tetrazolium bromide (MTT), as well as all other compounds, unless otherwise noted, were obtained from Sigma-Aldrich. Formulations were available on the market: Roundup GT+ (approval 2020448), Matin EL (2020328), Starane 200 (8400600), Pirimor G (7500569), Confidor (9200543), Polysect Ultra SL (2080018), Maronee (2000420), Opus (9200018), and Eyetak (9400555). MTT was prepared as a 5 mg/mL stock solution in phosphate-buffered saline, filtered through a 0.22 *μ*m filter before use, and diluted to 1 mg/mL in a serum-free medium.

### 2.2. Cell Lines and Treatments

The human embryonic kidney 293 cell line (HEK 293, ECACC 85120602) was provided by Sigma-Aldrich (Saint-Quentin Fallavier, France). The hepatoma cell line HepG2 was provided by ECACC (85011430). JEG3 cell line (ECACC 92120308) was provided by CERDIC (Sophia-Antipolis, France). Cells were grown in phenol red-free EMEM (Abcys, Paris, France) containing 2 mM glutamine, 1% nonessential amino acid, 100 U/mL of antibiotics (a mixture of penicillin, streptomycin, and fungizone) (Lonza, Saint Beauzire, France), 10 mg/mL of liquid kanamycin (Dominique Dutscher, Brumath, France), and 10% Fetal Bovine Serum (PAA, les Mureaux, France). JEG3 cells were supplemented with 1 mM sodium pyruvate. Cells were grown with this medium at 37°C (5% CO_2_, 95% air) during 48 h to 80% confluence, then washed, and exposed 24 h with serum-free EMEM to the APs or the formulations. Before treatment, all the pesticides were solubilized in a 100% DMSO solution, then diluted in serum-free medium to reach 0.5% DMSO (which had been previously proven not to be cytotoxic for the cells), and adjusted to a similar pH. This model was validated [29] and cytotoxic effects were similar in presence of serum but delayed by 48 h.

### 2.3. Cytotoxicity Measurement

After treatments, succinate dehydrogenase (SD) activity assay (MTT) [30] was applied as described previously [25]. Integrity of mitochondrial dehydrogenase enzymes indirectly reflects the cellular mitochondrial respiration. The optical density was measured at 570 nm using a Mithras LB 940 luminometer (Berthold, Thoiry, France). The bioluminescent ToxiLight bioassay (Lonza, Saint Beauzire, France) was applied for the membrane degradation assessment, by the intracellular adenylate kinase (AK) release in the medium; this is described as a necrosis marker [28]. Finally, the apoptotic cell death was evaluated with the Caspase-Glo 3/7 assay (Promega, Paris, France). Luminescence was measured using a Mithras LB 940 luminometer (Berthold, Thoiry, France). These methods were previously described [25].

### 2.4. Statistical Analysis

The experiments were repeated at least 3 times in different weeks on 3 independent cultures (*n* = 9). All data were presented as the means ± standard errors (SEMs). LC50 values were the best-fitted value of a nonlinear regression using sigmoid (5-parameter) equation with the GraphPad Prism 5 software. The differential effects between APs and formulations are measured by the surfaces between the curves by the calculation of integrals with ImageJ software [31]. Statistical differences of necrosis and apoptosis assays were calculated by a nonparametric Mann-Whitney test with the GraphPad Prism 5 software.

## 3. Results

**Figure 1:**
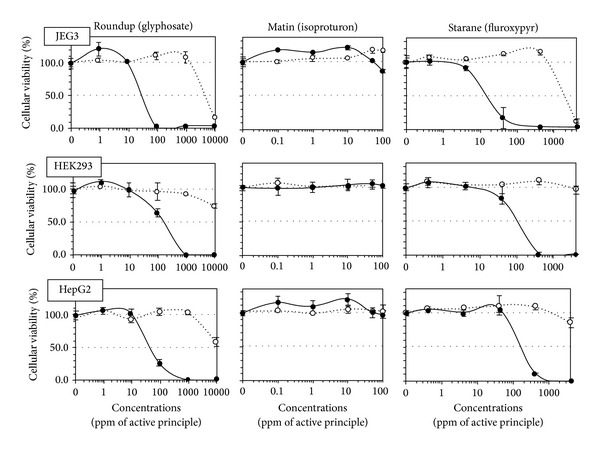
Differential cytotoxic effects between formulations of herbicides and their active principles (APs) on HepG2, HEK293, and JEG3 human cell lines. Effects on the mitochondrial succinate dehydrogenase (SD) activity, reflecting cell respiration inhibition, were measured in percentage of control in serum-free medium after 24 h of exposure. The concentrations in ppm are dilutions of each AP (dotted line) and their equivalent in formulation with adjuvants (solid line). All formulations are more toxic than their APs, except for isoproturon. SEMs are shown in all instances (*n* = 9).

**Figure 2:**
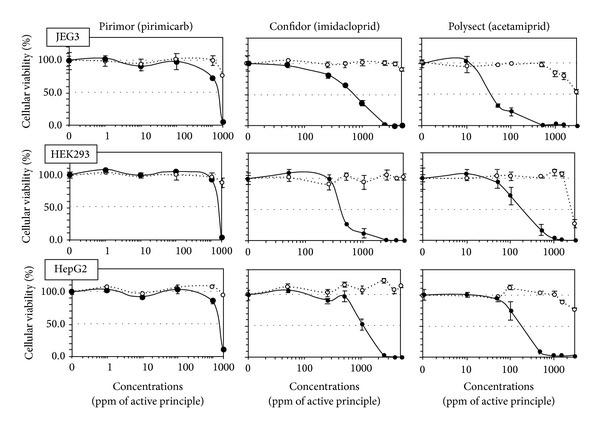
Differential cytotoxic effects between formulations of insecticides and their APs on HepG2, HEK293, and JEG3 human cell lines. The three described human cell lines were used in the conditions of Figure 1 and the results were almost identical. All formulations (solid line) are more toxic than their APs (dotted line); APs are slightly cytotoxic. SEMs are shown in all instances (*n* = 9).

**Figure 3:**
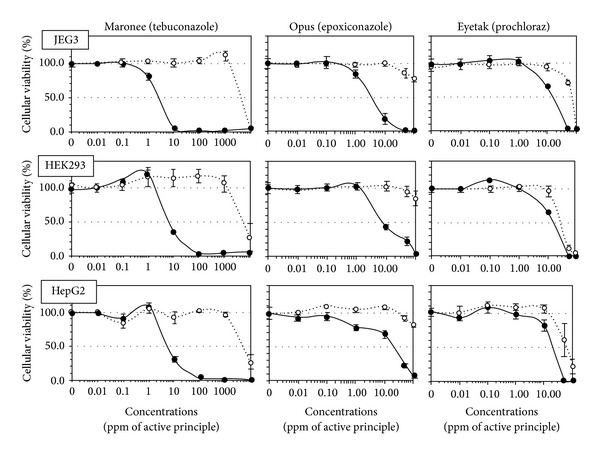
Differential cytotoxic effects between formulations of fungicides and their APs on HepG2, HEK293, and JEG3 human cell lines. The three described human cell lines were used in the culture conditions of Figure 1, and the results were almost identical. All formulations (solid line) are more cytotoxic than their APs (dotted line). Maronee is the most toxic compound tested from 1 ppm in JEG3. SEMs are shown in all instances (*n* = 9).

All formulations were cytotoxic and far more toxic than their APs, except for isoproturon and its formulated pesticide Matin which were both not soluble over 100 ppm. As a matter of fact, Matin does not have any declared adjuvant (Table 1). On human cells, among the tested products, fungicides were the most toxic (Figure 1), being cytotoxic from doses 300–600 times lower than agricultural dilutions, followed by herbicides (Figure 2) (except Matin) and then insecticides (Figure 3). JEG3 was the most sensitive cell line, the LC50 being on average, respectively, 7% and 23% lower than for HEK293 and HepG2, the least sensitive. The LC50 is calculated over 24 h. In all cell types, fungicides were the most toxic (mean LC50 12 ppm). They were followed by the herbicide Roundup (LC50 63 ppm), twice as toxic as Starane, and more than 10 times as toxic as the 3 insecticides, which represent the less toxic group (mean LC50 720 ppm). The APs of fungicides were the only APs that were toxic alone in our system, from 50 ppm in JEG3 for prochloraz, but they were still less toxic than their formulations.

In fact, 8 formulations out of 9 were clearly on average several hundred times more toxic than their APs, ranging from 2-3 times more toxic for pirimicarb or prochloraz to 1056 times more toxic for tebuconazole. Results were similar for all cell types.

**Figure 4:**
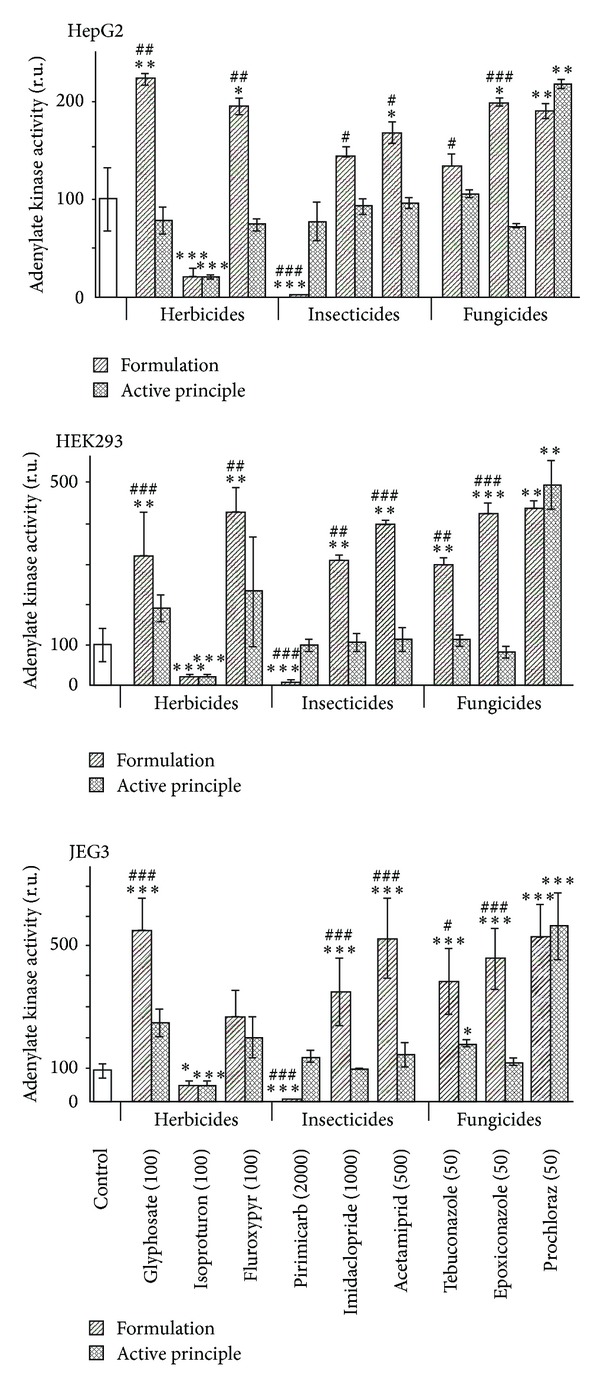
Differential necrotic effects between formulations and their APs. The three described human cell lines were used in the culture conditions of Figure 1. We have chosen the doses at the first differential effects measured by MTT assay. Formulations (stripped columns, expressed in ppm of the AP) are generally more cytotoxic than their APs (dashed columns) due to a necrotic effect of adjuvants. SEMs are shown in all instances (*n* = 9). For the comparison of each AP or formulation to the control (white column), *P < 0.05, **P < 0.01, and ***P < 0.001 in a nonparametric Mann-Whitney test. # symbol is used similarly for comparisons between APs and their formulations.

**Figure 5:**
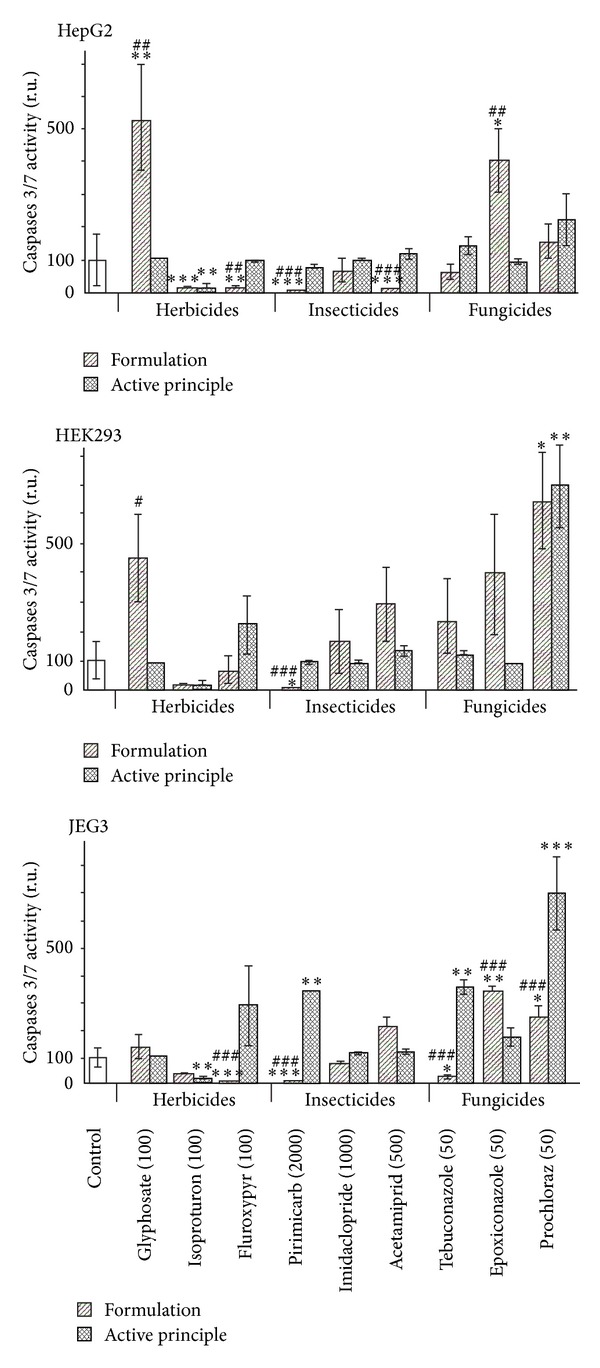
Differential apoptotic effects between formulations and their APs. The three described human cell lines were used in the culture conditions of Figure 1. We have chosen the doses at the first differential effects measured by MTT assay. SEMs are shown in all instances (*n* = 9). For the comparison of each AP or formulation to the control (white column), *P < 0.05, **P < 0.01, and ***P < 0.001 in a nonparametric Mann-Whitney test. # symbol is used similarly for comparisons between APs and their formulations.

This was even better understood by the differential measurement of the cytotoxicity through membrane disruption (Figure 4) or caspases activation (Figure 5). For the three cell lines, membrane disruptions are comparable. Most of the pesticides were necrotic and more necrotic than their APs except for Eyetak whose active principle prochloraz is the main toxicant of the formulation. We have not obtained relevant results with Pirimor because a green dye in the formulated product prevents the lecture of luminescence. Differential effects on apoptosis (Figure 5) were less obvious. With the formulated herbicides and insecticides, apoptosis levels are mostly decreased because of the prevailing effects of necrosis. This is not the case with fungicides which are apoptotic depending on the cell line. JEG3 cell lines are the most sensitive to apoptosis, in particular with fluroxypyr, pirimicarb, tebuconazole, and prochloraz. Overall, adjuvants in pesticides are thus far from inerts but cell membrane disruptors and induce in addition mitochondrial alterations.

## 4. Discussion

This is the first time that all these formulated pesticides have been tested on human cells well below agricultural dilutions. The three different cell types reacted very similarly and the toxicities were observed on several biomarkers; this confirmed our results. Moreover, these are very consistent with several studies on cell lines [1, 25], where placental JEG3 cells were found to be the most sensitive. In this study [1], adjuvants were also more cytotoxic through the disruption of membrane and mitochondrial respiration than from an activation of apoptotic pathways. Primary cells are in some case up to 100 times more sensitive, for instance, neonate umbilical cord vein cells [25]. We also study here short exposures (24 h), but we have previously demonstrated a time-amplifying effect: the differential toxicity between the AP glyphosate and Roundup is increased by 5 times in 72 h [29]. It appears that, with cell lines and short exposures, we underestimate by far the direct toxicity of the products in the long term. In this case in vivo, the metabolism may reduce the toxic effect, but this can be compensated or amplified by bioaccumulation and/or the combined effect of the AP with adjuvants. For instance, in this experiment, after 24 h, 63 ppm of Roundup was found to be toxic to cells, but in our previous experiment, after two years in rats, only 0.1 ppb of Roundup was found to be sufficient to provoke pathologies [2].

Adjuvants in pesticides are generally declared as inerts, and for this reason they are not tested in long-term regulatory experiments. It is thus very surprising that they amplify up to 1000 times the toxicity of their APs in 100% of the cases where they are indicated to be present by the manufacturer (Table 1). In fact, the differential toxicity between formulations of pesticides and their APs now appears to be a general feature of pesticides toxicology. As we have seen, the role of adjuvants is to increase AP solubility and to protect it from degradation, increasing its half-life, helping cell penetration, and thus enhancing its pesticidal activity [32] and consequently side effects. They can even add their own toxicity [1]. The definition of adjuvants as “inerts” is thus nonsense; even if the US Environmental Protection Agency has recently changed the appellation for “other ingredients,” pesticide adjuvants should be considered as toxic “active” compounds.

In the scientific literature, in contrast with regulatory beliefs, some harmful effects of the adjuvants present in this study are reported. In the formulations (Table 1) Starane 200, Opus, and Eyetak, the adjuvants include solvent naphtha (a petroleum distillate), which is known to have developmental effects in rodents [33]. Xylene (in Eyetak) has long been associated with cardiac and central nervous system diseases in humans [34]. 1-Methyl-2-pyrrolidinone (in Confidor) is a developmental toxicant and caused malformations, incomplete ossification of skull, and decreased fetal body weights in rats [35]. N,N-Dimethyldecanamide (Maronee adjuvant) has been characterized as a developmental toxicant in rodents [36] but is insufficiently studied for reproductive toxicity. The distinction between AP and “declared inert” compounds appears to be a regulatory assumption with no toxicological basis, from this experiment and others. Even industry and regulators contradict themselves in the classification of APs and inert compounds. For example, 1,2-benzisothiazoline-3-one is classed as an inert ingredient in the pesticide Polysect in particular and as an active ingredient in cleaning products [37].

All this does not exclude the toxicity of APs alone. Glyphosate inserted in the aromatase active site of mammalian cells disrupts steroidogenesis [23]. Imidacloprid alters the developing immunity in rats [38]. Fluroxypyr (ester 1-methylheptyl) has never been tested in human cells before this study but appears to be toxic from 22 ppm in formulation; its ADI is only 0.8 ppm/day (DG SANCO, 2013). It also appears here that prochloraz is the main toxicant of the tested formulation.

It is commonly believed that Roundup is among the safest pesticides. This idea is spread by manufacturers, mostly in the reviews they promote [39, 40], which are often cited in toxicological evaluations of glyphosate-based herbicides. However, Roundup was found in this experiment to be 125 times more toxic than glyphosate. Moreover, despite its reputation, Roundup was by far the most toxic among the herbicides and insecticides tested. This inconsistency between scientific fact and industrial claim may be attributed to huge economic interests, which have been found to falsify health risk assessments and delay health policy decisions [41].

In conclusion, our results challenge the relevance of the ADI, because it is calculated today from the toxicity of the AP alone in vivo. An “adjuvant factor” of at least a reduction by 100 can be applied to the present calculation of the ADI if this is confirmed by other studies in vivo. As an example, the present ADI for glyphosate is 0.3 ppm; for glyphosate-based herbicides it would be 3 ppb or less. However, this will never replace the direct study of the commercial formulation with its adjuvants in regulatory tests. Anyway, an exposure to a single formulated pesticide must be considered as coexposure to an active principle and the adjuvants. In addition, the study of combinatorial effects of several APs together may be very secondary if the toxicity of the combinations of each AP with its adjuvants is neglected or unknown. Even if all these factors were known and taken into account in the regulatory process, this would not exclude an endocrine-disrupting effect below the toxicity threshold. The chronic tests of pesticides may not reflect relevant environmental exposures if only one ingredient is tested alone.

## Figures and Tables

**Table 1:** Summary of the pesticides tested. We have tested 9 APs of major herbicides, insecticides, or fungicides of different classes, used worldwide for agricultural or domestic purposes. Concentrations of the APs are indicated in parenthesis. Adjuvants are reported where they are mentioned on the material safety data sheet (MSDS).

	Pesticide classes	Active principles	(g/L)	Formulations	Declared adjuvants
Herbicides	Phosphonoglycine	Glyphosate	450	Roundup GT+	Ethoxylated etheralkylamine
	Phenylurea	Isoproturon	500	Matin EL	Unknown
	Synthetic auxin	Fluroxypyr (ester 1-methylheptyl)	200	Starane 200	Solvent naphtha; alkyl-aryl sulfonates

Insecticides	Carbamate	Pirimicarb	500	Pirimor G	Docusate sodium; benzenesulfonic acid
	Neonicotinoid	Imidacloprid	200	Confidor	1-Methyl-2-pyrrolidinone
	Neonicotinoid	Acetamiprid	5	Polysect Ultra	1,2-Benzisothiazoline-3-one; ethanol

Fungicides	Triazole	Tebuconazole	250	Maronee	N,N-Dimethyldecanamide
	Triazole	Epoxiconazole	125	Opus	Solvent naphtha; fatty alcohol ethoxylated
	Imidazole	Prochloraz	450	Eyetak	Solvent naphtha; xylene; isobutanol
